# Acute Toxicity, Antioxidant, and Antifatigue Activities of Protein-Rich Extract from *Oviductus ranae*

**DOI:** 10.1155/2018/9021371

**Published:** 2018-02-25

**Authors:** Yang Zhang, Yang Liu, Kun Zhu, Yao Dong, Hao Cui, Liping Mao, Xiaoxiao Xu, Hongli Zhou

**Affiliations:** ^1^School of Chemistry and Pharmaceutical Engineering, Jilin Institute of Chemical Technology, Jilin 132022, China; ^2^Jilin Engineering Research Center for Agricultural Resources and Comprehensive Utilization, Jilin Institute of Chemical Technology, Jilin 132022, China; ^3^Department of Pharmacy, China-Japan Union Hospital of Jilin University, Changchun 130013, China; ^4^School of Biology and Food Engineering, Jilin Institute of Chemical Technology, Jilin 132022, China

## Abstract

The paper investigated the preparation, amino acid composition, acute toxicity, and *in vitro* and *in vivo* antioxidant, coupled with *in vivo* antifatigue activities of protein-rich extract of *Oviductus ranae* (PEOR). The results indicated that PEOR possesses high-safety property with maximum tolerated dose (MTD) higher than 20 g/kg in mice, shows weak scavenging capacities against hydroxyl, superoxide anion, and 1,1-diphenyl-2-picrylhydrazyl (DPPH) radicals, as well as ferric-reducing antioxidant power *in vitro*, but exerts strong antioxidant effect in ethanol-induced oxidative stress mice model; it can decrease malonaldehyde (MDA) and protein carbonyl (PCO) formation and increase total superoxide dismutase (T-SOD) activity and glutathione (GSH) synthesis. Besides the strong *in vivo* antioxidant activity, PEOR in a dose of 400 mg/kg also has antifatigue effect in mice, and it can prolong the exhaustive swimming time, reduce the elevated blood urea nitrogen (BUN) and blood lactic acid (BLA) caused by intense exercise. The *in vivo* activity of PEOR may be contributed by its absorbed amino acids, due to the fact that eight antioxidant amino acids and twelve glucogenic ones were found in it. This study will provide an evidence for the clinical use of PEOR as a dietary supplement for antioxidant and antifatigue in the same oral dose (400 mg/kg).

## 1. Introduction

Some harmful factors including overconsumption of drinking and smoking, X-ray irradiation, organic pollutants, and heavy metals can cause the overproduction of reactive oxygen species (ROS), which subsequently destroy the dynamic equilibrium between ROS generation and elimination to induce oxidative stress [1]. Moreover, under the physiological condition of oxidative stress, excess ROS can directly react with protein and DNA, as well as lipid to damage their structures and functions [2], leading to cell death and aging [3], coupled with some diseases, such as inflammation [4], immune deficiency [5], Parkinson's disease [6], Alzheimer's disease [7], and cancer [8]. Due to the fact that levels or activities of endogenous antioxidants, such as glutathione (GSH), superoxide dismutase (SOD), glutathione peroxidase (GPx), and catalases (CAT), are always lower than the level demanded for free radical-scavenging, it is usually necessary to supplement exogenous antioxidant when facing oxidative stress [9]. Another critical role of antioxidant lies in its positive effects on chronic fatigue syndrome (CFS) [10]. An increasing number of papers have demonstrated that antioxidant also exerts antifatigue activity *in vivo*, especially for some natural products [11–14].

Proteins are a kind of macromolecular substances consisting of different amino acids, which were found throughout most organs and tissues in the body. In addition to innate physiological functions comprising biological catalysis, DNA replication, muscle contraction, and molecules transportation [15], they also exhibit diverse biological activities, and antioxidant activity is one of the beneficial functions for human beings [16–18].

The Chinese brown frog, *Rana chensinensis*, is one of the famous economic animals farmed in China. It belongs to small amphibious frog with the mature male body size of 52~64 mm and female of 58~64 mm [19]. The natural populations of *R. chensinensis* mainly distribute in wet woodlands and mountains at low altitude ranging from 600 m to 1300 m in Northeastern China. In 1987, the wild *R. chensinensis* was listed as one of the national key-protected wild medicinal materials by the Chinese government, and its artificial breeding achieved large-scale reproduction in the 1990s [20, 21]. The economic value of *R. chensinensis* mainly depends on its dried oviduct, *Oviductus ranae* (OR), a traditional Chinese medicine (TCM) used in China for hundreds of years. OR was originally described in Compendium of Materia Medica in the Ming dynasty and listed in Chinese Pharmacopeia since 1985 [22]. Traditionally, OR was consumed as a tonic for the remedies of debilitation, insomnia, neurasthenia, respiratory symptoms, and climacteric syndrome [23]. Modern pharmacological studies have revealed that OR displays a wide range of activities including immune-enhancement [24], antiaging [25], antifatigue [26], antioxidation [27], antiosteoporosis [28], and estrogen-like effects [29]. OR is composed of proteins, lipids, steroids, vitamins, nucleic acids, and trace elements [30]; among them, proteins are the main constituents present in it; in most cases, their contents are more than 50% [24].

A great number of basic and experimental studies regarding oxidative stress have been performed to reveal the probable mechanisms involving the regulation of the imbalance between prooxidant and antioxidant system. These mechanisms provided major insights into oxidative stress and have advanced the clinical trials and approaches, resulting in successful prevention and diagnosis, as well as therapies [31]. Many natural antioxidants based on plants and other living organisms have been scientifically confirmed as effective therapeutic agents. Moreover, with the increase of healthy lifestyle pursuit, more and more people have consumed the natural antioxidants routinely. Furthermore, some individuals, especially athletes and sport professionals, are also eager for the antioxidant-possessing antifatigue functions. To date, numerous papers have reported the antioxidant activities of extracts or isolated compounds from TCM, which has become one of the most abundant sources of novel antioxidant discovery [32]. It is therefore important to obtain efficient natural antioxidants that can also exert antifatigue activities without any damages on the healthy consumers from TCM. OR is a precious TCM with high protein content, but, to our knowledge, there is little information on the antioxidant-related activities of proteins in it. Thus, in present study, with the aim at obtaining the potential of proteins from OR for becoming an antioxidant supplement with high-safety property. The protein-rich extract from OR (PEOR) was prepared and analyzed, and the acute toxicity and in *vitro* and in *vivo* antioxidant activities of PEOR were assessed. Then the in *vivo* antifatigue effect of PEOR was further evaluated.

## 2. Materials and Methods

### 2.1. Materials

OR samples were obtained from Jilin Huangzhihua Pharmaceutical Co. Ltd (Jilin, Changchun, China) and identified by Prof. Guangshu Wang, School of Pharmaceutical Sciences, Jilin University (Jilin, Changchun, China). Specimen of OR (voucher number HG-2035) was preserved in Jilin Engineering Research Center for Agricultural Resources and Comprehensive Utilization, Jilin Institute of Chemical Technology (Jilin, Jilin, China).

Ginsenoside (2.5 mg of Rg3 per 100 mg) was from Dalian Fusheng Pharmaceutical Co. Ltd (Liaoning, Dalian, China). The bovine serum albumin (BSA) was purchased from Nanjing Jiancheng Biotechnology Co. Ltd (Jiangsu, Nanjing, China). The majority of chemicals used for the *in vitro* antioxidant evaluation were obtained from Civi Chemical Technology Co. Ltd (Shanghai, China) including 1, 10-phenanthroline, vitamin C (VC), 1, 1-diphenyl-2-picrylhydrazyl (DPPH), nicotinamide adenine dinucleotide (NADH), nitroblue tetrazolium (NBT), and phenazine methosulphate (PMS). Other solvents and reagents were analytical grade and provided by Sigma Aldrich Chemical Co. Ltd (St. Louis, MO, USA).

Amino acid mixture standard solution including glycine, L-alanine, L-cystine, L-methionine, L-leucine, L-isoleucine, L-valine, L-glutamic acid, L-aspartic acid, L-phenylalanine, L-arginine, L-lysine, L-threonine, L-histidine, L-tyrosine, L-serine, L-proline, and ammonium chloride was obtained from Wako Pure Chemical Industries Ltd (Tokyo, Japan).

Biochemical indicators including aspartate transaminase (AST), alanine transaminase (ALT), glucose (GLU), triglycerides (TG), creatinine (CRE), and blood urea nitrogen (BUN) were determined by an AU2700 Beckman coulter chemistry analyzer (Beckman Coulter, Brea, CA, USA).

Reagent kits for the determination of malonaldehyde (MDA), total superoxide dismutase (T-SOD), glutathione (GSH), protein carbonyls (PCO), BUN and hepatic glycogen (HG) were obtained from Jiancheng Biotechnology Co. Ltd (Nanjing, Jiangsu, China).

Blood lactic acid (BLA) was determined by a Lactate Scout+ analyzer (EKF Diagnostics, Cardiff, WAL, England).

### 2.2. Preparation of PEOR

Prior to experiment, OR samples were grinded into power and sieved to 100 mesh. PEOR was prepared according to the reported method [24] with some modifications. About 10 g of OR powers were soaked in 1000 ml of phosphate buffered saline (PBS) solution (pH 6.5) at room temperature for 12 h, treated with ultrasonic wave at a power of 300 W for 2 h (Voshin, VS-200UE, Jiangsu, China). Then the mixture was centrifuged at 4500 rpm for 20 min at 4°C, the supernatant was filtered using a hollow-fiber membrane (0.45 *μ*m, GE Healthcare Life Sciences, Pittsburgh, PA, USA), and the precipitant was extracted twice as the above-mentioned method. The filtrate was mixed with ammonium sulfate (80% saturation) to produce precipitant, which was dissolved in distilled water and dialyzed for 24 h (MD10, Viskase, Darien, IL, USA). The dialysate was centrifuged at 4500 rpm for 20 min at 4°C, then the supernatant was freeze-dried, giving 1.02 g of PEOR, and the extraction yield was 10.2%.

### 2.3. General Chemical Analysis

Protein content of PEOR was determined by Bradford method using BSA as standard [33]. Moisture was determined via drying PEOR in an oven at 105°C for 6 h, and ash was determined by heating PEOR overnight at 550°C. Lipid content of PEOR was determined by Soxhlet method using petroleum ether as solvent [21, 34, 35].

### 2.4. Amino Acid Composition of PEOR

50 mg of PEOR was mixed with 5 ml of 6 N hydrochloric acid at 110 ± 1°C for 24 h under the protection of nitrogen atmosphere [36]. Before analysis, the pH value of hydrolysate solution was adjusted to 2.2 with 4 mol/l LiOH. Then the amino acid composition was analyzed by a fully automated amino acid analyzer (L-8900 Hitachi, Tokyo, Japan). The analytical conditions were as follows: chromatographic column, cation exchange resin 4.6 mm × 60 mm; temperature of column oven, 57°C; mobile phase, citric acid-sodium citrate at a flow rate of 0.25 ml/min; chromogenic agent, ninhydrin solution at a flow rate of 0.125 ml/min; temperature of derivatization, 135°C; sample size, 20 *μ*l; detection wavelength, 570 nm/440 nm.

### 2.5. Animals and Acute Toxicity of PEOR

#### 2.5.1. Experimental Animals

SPF-graded ICR mice (aged 4 weeks, weighing 20 ± 2 g, half male and half female) were purchased from the Experimental Animal Center of Jilin University (approval number SCXK (Ji) 2008-0005, Jilin, Changchun, China). Animals were feed in polypropylene cages and allowed free access to food and water. The rearing conditions were as follows: temperature of 20 ± 2°C, relative humidity of 60 ± 10%, and a 12 h-light/dark regime. Animal experiments were conducted based on the National Institutes of Health Guide for the Care and Use of Laboratory Animals (NIH publications number 8023, revised 1978) and approved by the Animal Care and Welfare Committee of Jilin Institute of Chemical Technology.

#### 2.5.2. Acute Toxicity of PEOR

Acute toxicity evaluation was conducted based on the guideline of Organization for Economic Cooperation and Development (OECD) for acute oral toxicity and previous work [21, 37] with some modifications. Eighty mice (half male and half female) were randomly divided into four groups (20 in each group, in each group 10 per sex); prior to administration, animals were fasted for 12 h and had free access to water. PEOR was dissolved in distilled water (3 ml/100 g BW) and orally treated to mice in doses of 5 g/kg BW (administration once), 10 g/kg BW (administration twice in 12 h), and 20 g/kg BW (administration three times in 24 h); mice in normal control (NC) group were orally treated with equal amount of distilled water. After a single dose administration, mortality and clinical signs associated with toxicity were observed and recorded daily for consecutive two weeks; body weight changes were measured before and after administration on the 14th day.

On day 14, after being weighed, animals were fasted for 12 h (free access to water) and anesthetized with pentobarbital sodium in a dose of 50 mg/kg BW intraperitoneally. Blood samples were collected from orbit into nonheparinized Eppendorf tubes for the determination of serum biochemical parameters including AST (substrate method), ALT (substrate method), GLU (hexokinase method), TG (GPO-PAP method), CRE (sarcosine oxidase method), and BUN (urease/glutamate dehydrogenase method) using an AU2700 Beckman coulter chemistry analyzer (Beckman Coulter, Brea, CA, USA). Then animals were euthanized with carbon dioxide, and a complete necropsy was performed. Some vital organs comprising liver, spleen, kidney, and testes/ovaries were harvested and weighed. Relative organ weight was calculated according to the following formula:

(1) $$\begin{array}{c} Relative\;organ\;weight\left(\%\right)=\frac{organ\;weight}{body\;weight}\times 100. \end{array}$$

Organs collected from animals were preserved in formalin solution (10%, pH 7.4) for the further histopathologic examination.

### 2.6. In Vitro Antioxidant Activity of PEOR

#### 2.6.1. Hydroxyl Radical-Scavenging Assay

Hydroxyl radical-scavenging assay of PEOR was conducted based on the method reported by You et al. [38] with some modifications. PEOR samples were dissolved in distilled water to prepare solutions at different concentrations (2, 4, 6, 8, and 10 mg/ml). 2 ml of PEOR solution and 1 ml of PBS solution containing 0.75 mmol/l 1, 10-phenanthroline (pH 7.4) were mixed together. Subsequently, 1 ml of 0.75 mmol/l FeSO_4_ and 1 ml of H_2_O_2_ solution (0.12%, *v*/*v*) were added. After being incubated at 37°C for 60 min, the absorbance of mixture (A_s_) was determined at 536 nm using an UV-visible spectrophotometer (722 N, Jingke Scientific Instrument Co. Ltd., Shanghai, China). The other two reaction systems in the absence of H_2_O_2_ and PEOR samples were used as normal control (A_c_) and blank (A_0_) solutions, respectively. VC at concentrations of 0.01, 0.02, 0.03, 0.04, and 0.05 mg/ml were used as positive control. The hydroxyl radical-scavenging rate was calculated as the following formula:

(2) $$\begin{array}{c} Hydroxyl\;radical-scavenging\;rate\left(\%\right)=\left(A_{s}-A_{0}\right)\times \frac{100}{\left(A_{c}-A_{0}\right)}. \end{array}$$

#### 2.6.2. DPPH Radical-Scavenging Assay

DPPH radical-scavenging activity of PEOR was determined using the previously reported method [39] with some modifications. PEOR samples were dissolved in distilled water to prepare solutions at concentrations of 2, 4, 6, 8, and 10 mg/ml. 2 ml of PEOR solution and 2 ml of 0.1 mmol/l DPPH ethanol solution were mixed and reacted in the dark for 30 min at room temperature. Then the absorbance of the mixture was measured at 517 nm (A_s_). The reaction system in the absence of DPPH was used as normal control (A_c_), system in the absence of PEOR used as blank solution (A_0_). VC at concentrations of 0.02, 0.04, 0.06, 0.08, and 0.1 mg/ml was used as positive control. The DPPH radical-scavenging rate was calculated by the following equation:

(3) $$\begin{array}{c} DPPH\;radical-scavenging\;rate\left(\%\right)=\left(A_{s}-A_{c}\right)\times \frac{100}{A_{0}}. \end{array}$$

#### 2.6.3. Superoxide Anion Radical-Scavenging Assay

Superoxide anion radical-scavenging activity of PEOR was assessed by the method reported by Li et al. [40] with some modifications. PEOR samples were dissolved in distilled water to prepare solutions at concentrations of 1, 2, 3, 4, and 5 mg/ml. 1 ml of PEOR solution and 3 ml of Tris-HCl buffer (16 mmol/l, pH 8.0) containing 0.5 ml of NADH solution (470 *μ*mol/l) and 0.5 ml of NBT solution (300 *μ*mol/l) were mixed, and then 0.5 ml of PMS solution (60 *μ*mol/l) was added to start the reaction. After being incubated at room temperature for 5 min, the absorbance of the mixture was read at 560 nm (A_s_), mixture without PEOR samples was used as blank control (A_0_). VC at concentrations of 0.01, 0.02, 0.03, 0.04, and 0.05 mg/ml was used as positive control. The superoxide anion radical-scavenging rate was estimated by the following equation:

(4) $$\begin{array}{c} Superoxide\;anion-scavenging\;rate\left(\%\right)=\left(A_{0}-A_{s}\right)\times \frac{100}{A_{0}}. \end{array}$$

#### 2.6.4. Reducing Power Assay

Reducing power was assayed according to the method reported by Wang et al. [34] with some modifications. Different concentrations (8, 10, 12, 14, and 16 mg/ml) of PEOR solutions were prepared. 1 ml of PEOR solution, 2.5 ml of phosphate buffer (0.2 mol/l, pH 6.6), and 2.5 ml of potassium ferricyanide solution (1%, *w*/*v*) were mixed and incubated at 50°C for 20 min; 2.5 ml of trichloroacetic acid (10%, *v*/*v*) was added. The mixture was centrifuged at 3000 rpm for 10 min, 2.5 ml of supernatant was mixed with 2.5 ml of distilled water and 0.5 ml of ferric chloride solution (0.1%, *w*/*v*), and then the absorbance at 700 nm was measured. VC at concentrations of 0.01, 0.02, 0.03, 0.04, and 0.05 mg/ml was used as positive control.

### 2.7. In Vivo Antioxidant Activity of PEOR

Sixty male ICR mice (aged 4 weeks, weighing 20 ± 2 g) were randomly divided into six groups (10 mice per group) as follows: normal control (NC), positive control (PC), oxidative stress model control (MC), and three PEOR-treated groups. Prior to experiment, animals were fasted for 12 h. Mice in PEOR-treated groups were administered with PEOR solution (2 ml/100 g BW) by oral gavage once a day in doses of 100, 200, and 400 mg/kg BW for 30 consecutive days, mice in PC group were treated with VC in a dose of 200 mg/kg BW, and mice in NC and MC groups were dosed with equal amount of distilled water. Dose selection of PEOR was based on the results of preliminary test (data not shown).

On the last day, after being fasted for 12 h (free access to water), except the mice in NC group, others were orally administered with a solution of 50% (*v*/*v*) ethanol to induce oxidative stress in a dose of 12 ml/kg BW. After 6 h, animals were anesthetized with pentobarbital sodium, and blood samples were collected from orbit to prepare serum for the determination of T-SOD and MDA by being centrifuged at 4°C, 4000 rpm for 10 min. Then animals were euthanized with carbon dioxide, livers were immediately dissected, washed, homogenized in physiological saline, and centrifuged at 4°C, 4000 rpm for 10 min to obtain supernatant for the quantification of GSH and PCO. The MDA (thiobarbituric acid method), T-SOD (hydroxylamine method), GSH (spectrophotometric method), and PCO (spectrophotometric method) levels were determined according to the methods described in the instructions of kits (Jiancheng Biotechnology Co. Ltd, Nanjing, Jiangsu, China) [41, 42].

### 2.8. In Vivo Antifatigue Activity of PEOR

The *in vivo* antifatigue evaluation of PEOR was designed and performed according to our previous work and reported method [13, 43, 44] with some modifications.

#### 2.8.1. Experimental Design

**Figure 1:**
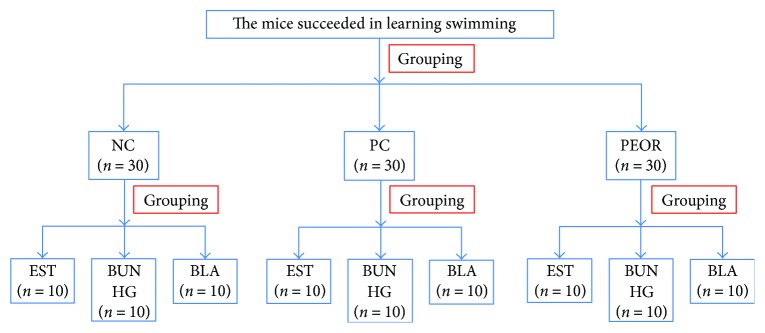
The flow chart for experimental design of antifatigue evaluation of PEOR. NC: normal control (distilled water); PC: positive control (ginsenoside, 50 mg/kg BW); PEOR (400 mg/kg BW); EST: exhaustive swimming test; BUN: blood urea nitrogen; HG: hepatic glycogen; BLA: blood lactic acid.

Before experiment, male ICR mice (aged 4 weeks, weighing 20 ± 2 g) swam twice a day (10 min each time) within one week to accustom themselves to swimming; mice that failed to learn swimming were eliminated. Then 90 animals were randomly divided into three groups (30 animals each) as normal control (NC), positive control (PC), and PEOR-treated group. After being fasted for 12 h, mice in NC group were orally administered with distilled water, mice in PC group were treated with ginsenoside (2.5 mg Rg3 per 100 mg) in a dose of 50 mg/kg BW, and mice in PEOR-treated group were administered with PEOR in a dose of 400 mg/kg BW once a day for 30 consecutive days. Then the animals in each group were further divided into three subgroups of 10 mice each according to following three test/determination section (Figure 1).

#### 2.8.2. Exhaustive Swimming Test

On the last day, animals were allowed to rest for 30 min after oral gavage, and ten mice in each group were attached to the tail of a tin wire (about 5% of body weight). Then mice were placed in a plastic swimming pool (50 cm × 50 cm × 40 cm) with temperature of 25 ± 1°C and depth of 30 cm. Exhaustive swimming time was recorded as the time when animals failed to rise to the surface to breathe within 10 s.

#### 2.8.3. Determination of BUN and HG

After the last administration, mice in the second subgroup were allowed to rest for 30 min then forced to swim without load. After swimming for 30 min, animals were anesthetized with pentobarbital sodium, and blood samples were collected from orbit to prepare serum for the quantification of BUN using kit (urease-Berthelot method, Jiancheng Biotechnology Co. Ltd, Nanjing, Jiangsu, China). Then mice were euthanized with carbon dioxide, and livers were harvested and homogenized for the determination of HG using kit (spectrophotometric method, Jiancheng Biotechnology Co. Ltd, Nanjing, Jiangsu, China).

#### 2.8.4. Determination of BLA

Mice in the third subgroup were also subjected to a forced swimming without load, before swimming, blood samples were taken from the eyeball for the quantification of BLA (C_1_) using a Lactate Scout+ analyzer (EKF Diagnostics, Cardiff, WAL, England). After swimming for 30 min, another blood samples were immediately collected for the determination of BLA (C_2_), and after resting for 20 min, blood samples were taken again for the determination of BLA (C_3_). The area under the curve of BLA (AUC_BLA_) was calculated as the following formula:

(5) $$\begin{array}{c} {AUC}_{BLA}\left(\frac{mmol}{l}\right)=5\times \left(C_{1}+3\times C_{2}+2\times C_{3}\right). \end{array}$$

### 2.9. Statistical Analysis

Experimental data was expressed as mean ± SD (standard deviation), and statistical analysis was performed using a SPSS19.0 software (SPSS Inc., Chicago, USA). For results of the *in vitro* evaluation, *t*-test was used to evaluate the significance of distances between two means, and for results of the *in vivo* evaluation, Levene's test was used to detect the homogeneity of variances, if homogeneous, one-way analysis of variance (ANOVA) was operated.

## 3. Results

### 3.1. General Chemical Analysis

The total protein content of PEOR was found to be 80.35 ± 2.71%. In addition, PEOR contains 2.64 ± 0.15% lipids, 7.41 ± 0.28% ash, and 1.12 ± 0.06% moisture.

### 3.2. Amino Acid Analysis of PEOR

**Figure 2:**
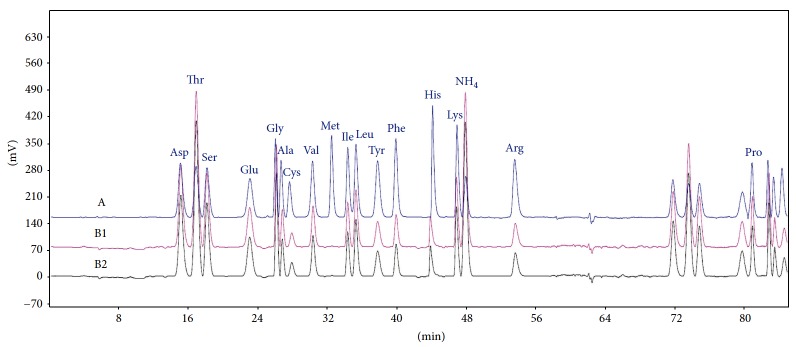
Chromatograms of amino acid standard mixture (A) and PEOR sample (B1 and B2). Asp: aspartic acid; Thr: threonine; Ser: serine; Glu: glutamic acid; Gly: glycine; Ala: alanine; Cys: cystine; Val: valine; Met: methionine; Ile: isoleucine; Leu: leucine; Tyr: tyrosine; Phe: phenylalanine; His: histidine; Lys: lysine; Arg: arginine; Pro: proline. Two parallel injections of sample.

The chromatograms of amino acid standard mixture and PEOR sample were shown in Figure 2, and amino acid composition of PEOR was summarized in Table 1. Seventeen amino acids were noted in PEOR, seven of them were essential amino acids, which accounted for 41.9%. The top three amino acids present in PEOR were threonine, aspartic acid, and serine with threonine as the highest content of 120 mg/g.

### 3.3. Acute Toxicity of PEOR

During the period of 14 days, no death and noticeable clinical signs associated with toxicity were found in NC and all PEOR-treated groups.

#### 3.3.1. Body Weight

As shown in Table 2, the body weights of mice increased gradually during the study period, when compared with NC group, no significant differences in body weight changes were observed.

#### 3.3.2. Relative Organ Weight

The effects of PEOR on relative weight of vital organs including liver, kidney, spleen, and testis/ovary were demonstrated in Table 3. No significant differences in relative organ weight were noted between NC and PEOR-treated groups.

#### 3.3.3. Biochemical Parameter

Some biochemical parameters (AST, ALT, GLU, TG, CRE, and BUN) reflecting the pathological changes of vital organs were determined, and results were shown in Table 4. Statistical analysis of these parameters indicated that there were no significant differences between NC and PEOR-treated groups.

#### 3.3.4. Histopathological Examination

**Figure 3:**
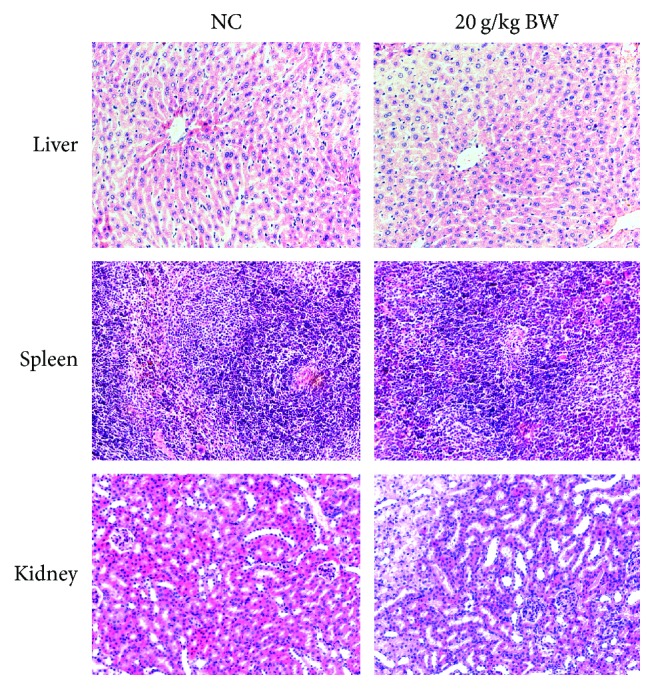
Representative microphotographs of the liver, spleen, and kidney of mice at 200× from NC and 20 g/kg BW of PEOR-treated groups.

The microphotographs of histopathological observation of liver, spleen, and kidney in NC and PEOR-treated groups were exhibited in Figure 3. When compared with NC group, any obvious tissue changes were not found.

### 3.4. In Vitro Antioxidant Activity of PEOR

**Figure 4:**
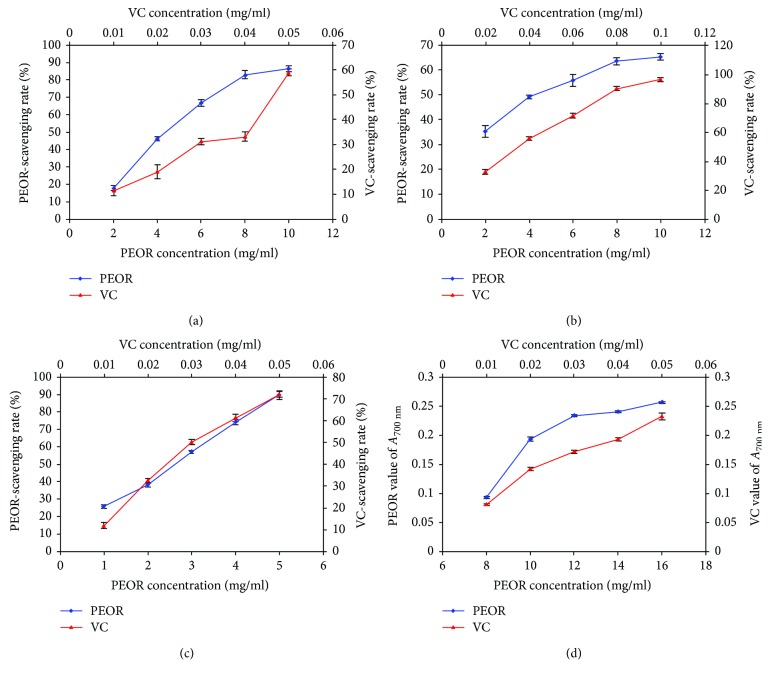
The *in vitro* antioxidant activities of PEOR using VC as a positive control. (a) Hydroxyl radical-scavenging activity; (b) DPPH radical-scavenging activity; (c) superoxide anion radical-scavenging activity; (d) reducing power. Data was expressed as the mean ± SD (*n* = 3).

The *in vitro* scavenging capacities of PEOR against hydroxyl, DPPH, and superoxide anion radicals, as well as ferric ion-reducing power, were shown in Figure 4, and the corresponding half inhibitory concentration (IC_50_) value was expressed in Table 5. In the range of 2~10 mg/ml, hydroxyl radical-scavenging activity of PEOR increased with the increase of sample concentration, the highest scavenging rate against hydroxyl radical of PEOR was 86.35 ± 1.82%, and the IC_50_ value was 4.85 ± 0.06 mg/ml, which was much lower than that of positive control (VC) with the IC_50_ value of 0.0476 ± 0.0005 mg/ml (Figure 4(a)). As for DPPH radical, in the range of 2~10 mg/ml, PEOR also displayed radical-scavenging activity, which increased with the elevation of PEOR concentration, and the highest scavenging rate was 65.23 ± 1.22%, but inferior to VC, the IC_50_ values of PEOR and VC against DPPH radical were 4.98 ± 0.37 mg/ml and 0.036 ± 0.0011 mg/ml, respectively (Figure 4(b)). In the range of 1~5 mg/ml, PEOR exhibited scavenging activity against superoxide anion radical in a good linear relationship to sample concentration (*R*^2^ = 0.9971), and the highest scavenging rate was 89.6 ± 2.43%, but its activity was still much lower than VC; the corresponding IC_50_ values were 2.58 ± 0.02 mg/ml and 0.0332 ± 0.0006 mg/ml, respectively (Figure 4(c)). In the range of 8~16 mg/ml, the absorbance (A) of PEOR at 700 nm increased with the increase of sample concentration. When *A*_700 nm_ was 0.2, the concentration of PEOR was 11.8 ± 0.02 mg/ml, and the corresponding concentration of VC was 0.0395 ± 0.0002 mg/ml (Figure 4(d)).

### 3.5. In Vivo Antioxidant Activity of PEOR

#### 3.5.1. Effects of PEOR on MDA

**Figure 5:**
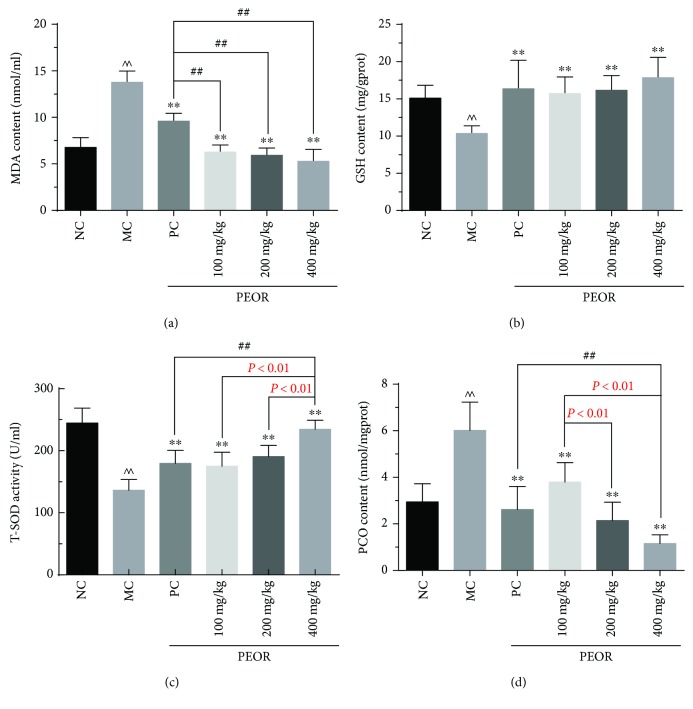
Effects of PEOR on (a) MDA, (b) GSH, (c) T-SOD, and (d) PCO. Data denoted were means + SD (*n* = 10). Different symbols indicate statistically significant differences, ^∧∧^*P* < 0.01 as compared with NC group; ^∗∗^*P* < 0.01 as compared with MC group; ^##^*P* < 0.01 as compared with PC group. MDA: malonaldehyde; GSH: glutathione; T-SOD: total superoxide dismutase; PCO: protein carbonyls; NC: normal control; MC: model control; PC: positive control (VC in a dose of 200 mg/kg BW).

As shown in Figure 5(a), MDA contents decreased with the increase of PEOR dose; the lowest content of MDA was 5.28 ± 1.27 mmol/l, 2.6-fold lower than that in MC, and 1.8-fold lower than that in PC group. Statistical analysis of MDA contents indicated that there were significant differences (*P* < 0.01) between NC and MC groups. When compared with MC group, significant differences (*P* < 0.01) were found in PC and all PEOR-treated groups. Significant differences (*P* < 0.01) were also noted compared with PC group.

#### 3.5.2. Effects of PEOR on GSH

As shown in Figure 5(b), when compared with NC, significant differences (*P* < 0.01) in GSH contents were observed in MC group, and significant differences (*P* < 0.01) were also found between MC and other groups. GSH contents increased with the increase of dose; when PEOR dose was 400 mg/kg, the content of GSH reached 17.85 ± 3.82 mg/gprot, which was a little higher than that in PC (16.36 ± 3.82 mg/gprot), but no statistically significant differences were noted.

#### 3.5.3. Effects of PEOR on T-SOD

As shown in Figure 5(c), T-SOD activities in MC decreased significantly (*P* < 0.01) compared with NC. When compared with MC, significant differences (*P* < 0.01) in T-SOD were found in PC and all PEOR-treated groups. Oral administration of PEOR can increase T-SOD activities in a dose-dependent manner (*P* < 0.01), when PEOR dose reached 400 mg/kg, the T-SOD activity was 234.6 ± 14.5 U/ml, which was significantly (*P* < 0.01) higher than that in PC (179.6 ± 21.0 U/ml).

#### 3.5.4. Effects of PEOR on PCO

As shown in Figure 5(d), PCO contents in MC group were significantly higher (*P* < 0.01) than those in NC. When compared with MC, significant differences (*P* < 0.01) in PCO were found in PC and all PEOR-treated groups. PCO contents decreased in a dose-dependent manner (*P* < 0.01), and significant differences (*P* < 0.01) were noted in 400 mg/kg of PEOR-treated group compared with PC (1.16 ± 0.37 nmol/mgprot versus 2.62 ± 0.99 nmol/mgprot).

### 3.6. In Vivo Antifatigue Activity of PEOR

#### 3.6.1. Exhaustive Swimming Test

**Figure 6:**
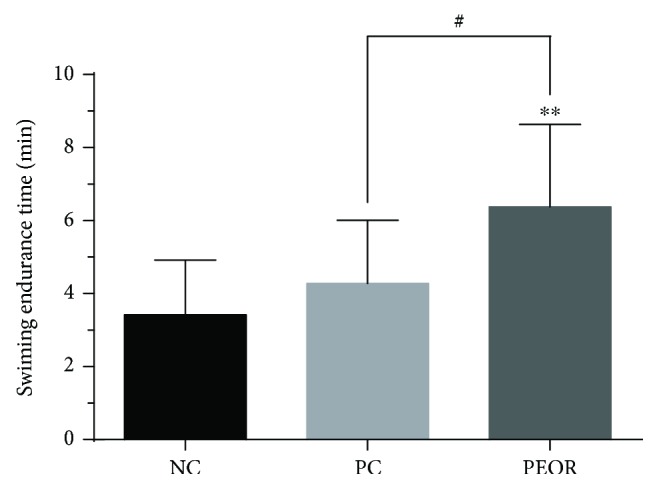
Effects of PEOR on exhaustive swimming endurance time. Data denoted were means + SD (*n* = 10). Different symbols indicate statistically significant differences, ^∗∗^*P* < 0.01 as compared with NC; ^#^*P* < 0.05 as compared with PC. NC: normal control; PC: positive control (ginsenoside in a dose of 50 mg/kg BW); PEOR (400 mg/kg BW).

As shown in Figure 6, there were significant differences (*P* < 0.01) between NC and PEOR-treated groups, and the swimming time was 6.38 ± 2.26 min, 1.9-fold longer than that in NC. When compared with PC, significant differences (*P* < 0.05) were found in PEOR-treated group, approximately 1.5-fold longer than that in PC.

#### 3.6.2. Effects of PEOR on BUN

**Figure 7:**
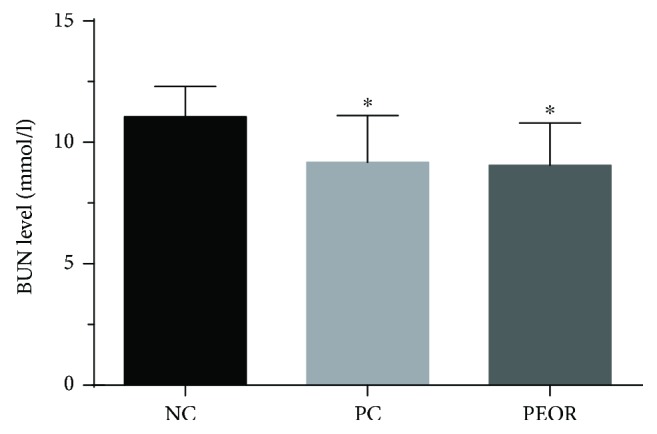
Effects of PEOR on BUN. Data denoted were means + SD (*n* = 10). Symbol indicates statistically significant differences, ^∗^*P* < 0.05 as compared with NC group. BUN: blood urea nitrogen; NC: normal control; PC: positive control (ginsenoside in a dose of 50 mg/kg BW); PEOR (400 mg/kg BW).

As shown in Figure 7, when compared with NC, BUN contents in PC and PEOR-treated groups were significantly (*P* < 0.05) lower by 18% and 17%, respectively.

#### 3.6.3. Effects of PEOR on HG

**Figure 8:**
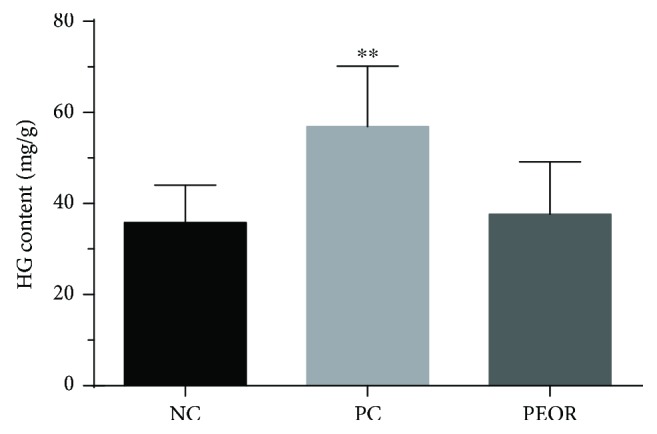
Effects of PEOR on HG. Data denoted were means + SD (*n* = 10). Symbol indicates statistically significant differences, ^∗∗^*P* < 0.01 as compared with NC group. HG: hepatic glycogen; NC: normal control; PC: positive control (ginsenoside in a dose of 50 mg/kg BW); PEOR (400 mg/kg BW).

As shown in Figure 8, HG contents in PEOR-treated group was 37.66 ± 11.49 mg/g and that in NC was 35.83 ± 11.49 mg/g; no significant differences were found. There were statistically significant differences (*P* < 0.01) in HG between PC and NC groups, the HG content in PC was 56.88 ± 13.3 mg/g, 1.59-fold higher than that in NC and 1.51-fold higher than that in PEOR-treated group.

#### 3.6.4. Effects of PEOR on BLA

BLA content at different time points (*t*_before swimming_, *t*_0 min after swimming_, and *t*_20 min after swimming_) was determined. As shown in Table 6, before swimming, no significant differences in BLA were noted among groups. When compared with NC group, at 0 min after swimming, the BLA contents in PC (*P* < 0.01) and PEOR-treated groups (*P* < 0.01) significantly decreased. After resting for 20 min, the elevated BLA levels of all groups were reduced, and no statistically significant differences were observed. The AUC_BLA_ value was calculated and found that there were significant differences (*P* < 0.05) in PC and PEOR-treated groups compared with NC group. The AUC_BLA_ value in PEOR-treated group was similar to that in PC group (115.4 ± 24.7 mmol/l versus 114.4 ± 19.4 mmol/l).

## 4. Discussion

Since *Oviductus ranae* (OR) is a precious TCM with abundant protein contents, thus, in this paper, the protein-rich extract of OR (PEOR) was prepared and analyzed. The results indicated that PEOR contains 80.35 ± 2.71% protein, which comprises seventeen amino acids, seven of them are essential amino acids with total contents of 41.9% (Figure 2 and Table 1).

In view of increasing number of therapeutic risks caused by the use of natural products [45–47] and our previous work [21], where we found that OR possesses high-safety property, in present study, only a single-dose oral toxicity with an observation of 14-day interval was conducted to evaluate the safety of PEOR, and 20 g/kg was taken as an upper limit dose. During the observation period, no death and noticeable clinical signs associated with toxicity were found in NC and PEOR-treated groups; there were also no significant changes in body weights (Table 2), suggesting that the maximum tolerated dose (MTD) of PEOR may be higher than 20 g/kg in mice.

Then a complete necropsy and serum biochemical and histopathological examinations were performed to assess the harmful effects of PEOR on inner organs. During necropsy, any noticeable abnormalities were not noticed, and there were no significant differences in relative weights of vital organs including liver, kidney, spleen, and testis/ovary between NC and PEOR-treated groups (Table 3). Serum biochemical parameter is another important profile to detect the *in vivo* injury degree of organs, for example, ALT and AST are closely related to the function of the liver, while CRE and BUN are important biomarkers of renal toxicity [48, 49]. When compared with NC, no significant differences in AST, ALT, GLU, TG, BUN, and CRE were found in PEOR-treated groups (Table 4), indicating that oral administration of PEOR has no harm on inner organs, which was further confirmed by the results of histopathological examination, where any obvious changes in liver, spleen, and kidney were not found compared with NC even in the maximum dose of 20 g/kg (Figure 3).

**Figure 9:**
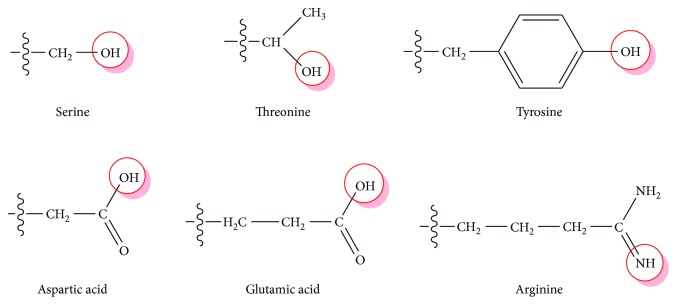
Hydrogen-donor side chains of amino acids present in PEOR.

In antioxidant assay, the *in vitro* evaluation was firstly conducted to obtain the antioxidant potential of PEOR. The results showed that PEOR exhibits certain scavenging capacities against hydroxyl, DPPH, and superoxide anion radicals, as well as certain reducing power to ferric ion in different concentrations, and activities increased with the increase of concentration (Figure 4 and Table 5). The free radical-scavenging activity of PEOR may be contributed by some of its amino acid residues, which may provide active hydrogen to destroy free radicals in liquid medium [50]; as shown in Table 1 and Figure 9 six amino acids with hydrogen-donor side chains were present in PEOR. However, when compared with VC, a well-known water-soluble antioxidant, the free radical-scavenging capacities and ferric ion-reducing power of PEOR were significantly (*P* < 0.01) lower; the IC_50_ values were about 100-fold higher than those of VC (Table 5). These disappointing results were consistent with the general finding that proteins or large polypeptides show lower free radical-scavenging capacities than their short peptides and amino acids, owing to the fact that smaller molecules are inclined to interact with free radicals more effectively [51]. In order to verify this hypothesis, the *in vivo* antioxidant evaluation of PEOR was further performed.

Based on the results of previous study [41], an ethanol-induced oxidative stress mice model was taken to evaluate the *in vivo* antioxidant activity of PEOR using VC in a dose of 200 mg/kg as positive control. Four antioxidant biomarkers including MDA (product of lipid peroxidation), GSH (endogenous antioxidant), T-SOD (antioxidase), and PCO (product of protein oxidation) were selected and determined. As shown in Figure 5, when compared with NC, significant differences (*P* < 0.01) in MDA, GSH, T-SOD, and PCO were found in MC, suggesting that an ethanol-induced oxidative stress mice model was well established. When compared with MC, significant differences (*P* < 0.01) in MDA, GSH, T-SOD, and PCO were noted in PEOR-treated groups, and the positive effects enhanced with the increase of dose; in the case of T-SOD and PCO, a dose-dependent (*P* < 0.01) manner was observed. When compared with PC, significant differences (*P* < 0.01) in MDA were found in all PEOR-treated groups, and similar tendencies (*P* < 0.01) in T-SOD and PCO were noted in 400 mg/kg of PEOR-treated group. These results revealed that oral administration of PEOR can reduce the oxidative stress caused by ethanol in mice and has more effects on MDA, T-SOD, and PCO than on GSH, especially for MDA. The 400 mg/kg in mice is a promising effective dose for antioxidant activity of PEOR. Its mechanism may involve the decrease of MDA and PCO formation and increase of T-SOD activity and GSH synthesis. The strong *in vivo* antioxidant activity of PEOR contradicted with the weak activity of *in vitro* evaluation, which further confirmed our speculation, that is, the absorbed amino acids and small peptides may be the real forms of PEOR to exert antioxidant effects. Previous studies have manifested that the types and compositions of absorbed protein digestive products are closely related to their activities [34], and some amino acids including threonine, cysteine, methionine, tryptophan, tyrosine, histidine, phenylalanine, glutamic acid, aspartic acid, and lysine show good antioxidant activities both *in vitro* and *in vivo* [52–54]. As shown in Table 1, there were eight antioxidant amino acids present in PEOR, that is, aspartic acid (69.4 mg/g), threonine (120 mg/g), glutamic acid (51.8 mg/g), methionine (1.13 mg/g), tyrosine (25.1 mg/g), phenylalanine (23.6 mg/g), histidine (13.9 mg/g), and lysine (42.0 mg/g), which accounted for approximately 52% of the total amount of amino acids present in PEOR; their existence may play an important role in the exertion of antioxidant effect *in vivo*.

Several findings have manifested that intense exercise-induced oxidative stress can cause the accumulation of free radicals and induce muscle fatigue [55, 56]. Exogenous dietary antioxidants can potentiate the scavenging effects of endogenous antioxidants to fight against fatigue [11]. In consideration of the strong *in vivo* antioxidant effects of PEOR, as well as its high safety, we then evaluated the *in vivo* antifatigue activity of PEOR in the promising dose found in antioxidant evaluation (400 mg/kg). In our previous work [43], we noticed that ginsenoside in a dose of 35 mg/kg in mice is a good positive control for the evaluation of antifatigue effect of natural product; in order to ensure the control full effectiveness, we slightly raised the dose and selected 50 mg/kg as the dose of positive control. The exhaustive swimming time, coupled with some biochemical indicators (BUN, HG, and BLA) reflecting fatigue degree, was determined to estimate the *in vivo* antifatigue effect of PEOR. As shown in Figures 6–8, PEOR in a dose of 400 mg/kg can prolong the exhaustive swimming time and reduce the elevated BUN content caused by intense exercise in mice, but little effect on HG storage. As for BLA profile (Table 6), PEOR has little effect on BLA under resting-state conditions but significantly (*P* < 0.01) reduces the elevated BLA level induced by intense exercise. When compared with PC (ginsenoside, 50 mg/kg), PEOR in a dose of 400 mg/kg can significantly (*P* < 0.05) improve the exercise tolerance, indicating that PEOR (400 mg/kg) is also a promising candidate for the development of nature-based antifatigue supplement, owing to the fact that exhaustive swimming time is the most direct and potent index to measure the antifatigue activity of tested sample [57]. The antifatigue capacity of PEOR could be also attributed to the extraenergy supplied by its glucogenic amino acids including aspartic acid (69.4 mg/g), threonine (120 mg/g), serine (53.3 mg/g), glutamic acid (51.8 mg/g), glycine (44.3 mg/g), alanine (30.8 mg/g), valine (28.5 mg/g), methionine (1.13 mg/g), isoleucine (28.1 mg/g), histidine (13.9 mg/g), arginine (25.5 mg/g), and proline (37.1 mg/g), which accounted for about 75% of the total amino acids present in PEOR (Table 1). The exact mechanism regarding antifatigue effect of PEOR deserved to be studied in the near future. In addition, due to the fact that most of the proteins enter the bloodstream as single amino acids [58, 59], in this paper, we mainly discussed the active contributions of amino acids in PEOR. Meanwhile, contributions of small peptides absorbed in intestinal tract to the antioxidant and antifatigue effects of PEOR, especially the chemical structures and activity-favourable conformations, need to be further explored.

## 5. Conclusion

In summary, PEOR is mainly composed of seventeen amino acids with seven essential ones. It possesses high safety with MTD value upper than 20 g/kg in mice and exerts weak scavenging capacities against hydroxyl, DPPH, and superoxide anion radicals, as well as ferric ion-reducing power *in vitro*, but exhibits strong antioxidant activity in ethanol-induced oxidative stress mice model; its mechanism may involve the decrease of MDA and PCO formation, associated with the increase of T-SOD activity and GSH synthesis. The *in vivo* antioxidant effect of PEOR increased with the increase of dose; 400 mg/kg is a promising dose deserved to be further studied; in this dose, PEOR also shows antifatigue effect. There are six amino acids with hydrogen-donor side chains, eight antioxidant amino acids, and twelve glucogenic amino acids present in PEOR; they may play an important role in exertion of the *in vitro* and *in vivo* antioxidant activity, as well as the *in vivo* antifatigue effect of PEOR.

## Figures and Tables

**Table 1:** Amino acid composition of PEOR.

Amino acid	Content (mg/g)	Percent composition (%)
Aspartic acid	69.4	10.4
^a^Threonine	120	18.0
Serine	53.3	7.98
Glutamic acid	51.8	7.75
Glycine	44.3	6.63
Alanine	30.8	4.61
Cystine	37.5	5.61
^a^Valine	28.5	4.27
^a^Methionine	1.13	0.17
^a^Isoleucine	28.1	4.21
^a^Leucine	36.4	5.45
Tyrosine	25.1	3.76
^a^Phenylalanine	23.6	3.53
Histidine	13.9	2.08
^a^Lysine	42.0	6.29
Arginine	25.5	3.82
Proline	37.1	5.55
Total	668	100

^a^Essential amino acid.

**Table 2:** Effects of PEOR on body weight in mice.

Sex	Group	Initial weight	Final weight	Weight gain
		(g)	(g)	(g)
Male	NC	19.0 ± 1.02	31.3 ± 2.27	12.3 ± 2.13
	5 g/kg	19.4 ± 1.29	30.8 ± 2.07	11.4 ± 1.78
	10 g/kg	19.1 ± 0.91	30.9 ± 1.93	11.8 ± 1.51
	20 g/kg	19.6 ± 1.42	31.1 ± 2.38	11.5 ± 1.87
Female	NC	18.2 ± 0.72	28.7 ± 1.87	10.5 ± 1.33
	5 g/kg	18.7 ± 0.94	28.4 ± 1.39	9.71 ± 0.78
	10 g/kg	19.1 ± 0.62	28.8 ± 2.12	9.65 ± 1.49
	20 g/kg	18.5 ± 0.79	29.2 ± 1.83	10.7 ± 1.24

No statistically significant differences were noted; NC: normal control; values are the means ± SD (*n* = 10).

**Table 3:** Effects of PEOR on relative organ weight in mice.

Sex	Group	Liver	Kidney	Spleen	Testis/ovary
		(%)	(%)	(%)	(%)
Male	NC	4.26 ± 0.49	1.33 ± 0.23	0.27 ± 0.05	0.51 ± 0.09
	5 g/kg	4.35 ± 0.53	1.29 ± 0.17	0.26 ± 0.07	0.52 ± 0.08
	10 g/kg	4.22 ± 0.57	1.31 ± 0.28	0.26 ± 0.05	0.53 ± 0.07
	20 g/kg	4.43 ± 0.54	1.30 ± 0.19	0.28 ± 0.04	0.49 ± 0.08
Female	NC	4.13 ± 0.38	1.17 ± 0.18	0.21 ± 0.04	0.038 ± 0.006
	5 g/kg	4.18 ± 0.42	1.15 ± 0.16	0.19 ± 0.05	0.042 ± 0.008
	10 g/kg	4.16 ± 0.37	1.16 ± 0.19	0.20 ± 0.06	0.039 ± 0.005
	20 g/kg	4.19 ± 0.43	1.12 ± 0.15	0.21 ± 0.06	0.041 ± 0.007

No statistically significant differences were noted; NC: normal control; values are the means ± SD (*n* = 10).

**Table 4:** Effects of PEOR on biochemical parameter in mice.

Sex	Group	AST	ALT	GLU	TG	CRE	BUN
		(U/l)	(U/l)	(mmol/l)	(mmol/l)	(*μ*mol/l)	(mmol/l)
Male	NC	143 ± 12.1	31.9 ± 4.08	4.53 ± 0.58	2.13 ± 0.08	45.2 ± 6.13	7.18 ± 0.55
	5 g/kg	142 ± 11.6	33.0 ± 3.34	4.41 ± 0.51	2.04 ± 0.17	47.6 ± 7.47	6.79 ± 0.46
	10 g/kg	141 ± 12.3	32.7 ± 3.93	4.49 ± 0.53	2.05 ± 0.09	43.9 ± 6.08	7.06 ± 0.68
	20 g/kg	142 ± 11.2	32.3 ± 4.22	4.51 ± 0.64	2.03 ± 0.07	45.8 ± 5.66	7.36 ± 0.71
Female	NC	118 ± 10.7	26.9 ± 3.52	4.38 ± 0.69	1.84 ± 0.12	39.6 ± 5.73	7.73 ± 0.91
	5 g/kg	117 ± 9.14	28.1 ± 3.83	4.75 ± 0.53	1.87 ± 0.28	37.2 ± 5.48	8.06 ± 0.54
	10 g/kg	121 ± 8.76	27.1 ± 2.89	4.63 ± 0.62	2.06 ± 0.15	36.8 ± 7.61	7.94 ± 0.62
	20 g/kg	118 ± 10.2	27.7 ± 3.23	5.06 ± 0.68	1.83 ± 0.26	38.4 ± 6.33	8.15 ± 1.23

No statistically significant differences were noted; NC: normal control; values are the means ± SD (*n* = 10).

**Table 5:** The IC_50_ values and reducing power of PEOR and VC.

Sample	IC_50_ (mg/ml)			Reducing power (mg/ml)^a^
	Hydroxyl radical	DPPH radical	Superoxide anion	
PEOR	4.85 ± 0.06^∗∗^	4.98 ± 0.37^∗∗^	2.58 ± 0.02^∗∗^	11.8 ± 0.02^∗∗^
VC	0.0476 ± 0.0005	0.036 ± 0.0011	0.0332 ± 0.0006	0.0395 ± 0.0002

^a^The corresponding concentrations of PEOR and VC, when *A*_700 nm_ = 0.2; data was expressed as the mean ± SD (*n* = 3); symbol indicates statistically significant differences, ^∗∗^*P* < 0.01 versus VC group.

**Table 6:** Effects of PEOR on BLA.

Group	Before swimming (C_1_)	0 min after swimming (C_2_)	20 min after swimming (C_3_)	Area under the curve (AUC_BLA_)
	(mmol/l)	(mmol/l)	(mmol/l)	(mmol/l)
NC	2.88 ± 0.79	6.38 ± 1.27	3.28 ± 0.67	142.8 ± 22.7
PC	2.72 ± 0.66	4.63 ± 0.94^∗∗^	3.13 ± 0.61	114.4 ± 19.4^∗^
PEOR	2.84 ± 0.84	4.59 ± 1.04^∗∗^	3.24 ± 1.17	115.4 ± 24.7^∗^

Data denoted were means ± SD (*n* = 10). Different symbols indicate statistically significant differences, ^∗^*P* < 0.05 and ^∗∗^*P* < 0.01 as compared with NC group. BLA: blood lactic acid; NC: normal control; PC: positive control (ginsenoside in a dose of 50 mg/kg BW); PEOR (400 mg/kg BW). AUC_BLA_ = 5 × (C_1_+ 3 × C_2_+ 2 × C_3_).
